# Molecular Biology of *Prune Dwarf Virus*—A Lesser Known Member of the *Bromoviridae* but a Vital Component in the Dynamic Virus–Host Cell Interaction Network

**DOI:** 10.3390/ijms18122733

**Published:** 2017-12-16

**Authors:** Edmund Kozieł, Józef J. Bujarski, Katarzyna Otulak

**Affiliations:** 1 Department of Botany, Faculty of Agriculture and Biology, Warsaw University of Life Sciences—SGGW, Nowoursynowska Street 159, 02-776 Warsaw, Poland; 2 Department of Biological Sciences, Northern Illinois University, DeKalb, IL 60115, USA; jbujarski@niu.edu; 3 Institute of Bioorganic Chemistry, Polish Academy of Sciences, Noskowskiego 12/14, 61-704 Poznań, Poland

**Keywords:** *Bromoviridae*, plant–virus interactions, plant defense response, *Prune dwarf virus*, replication process, systemic and local movement

## Abstract

*Prune dwarf virus* (PDV) is one of the members of *Bromoviridae* family, genus *Ilarvirus*. Host components that participate in the regulation of viral replication or cell-to-cell movement via plasmodesmata are still unknown. In contrast, viral infections caused by some other *Bromoviridae* members are well characterized. *Bromoviridae* can be distinguished based on localization of their replication process in infected cells, cell-to-cell movement mechanisms, and plant-specific response reactions. Depending upon the genus, “genome activation” and viral replication are linked to various membranous structures ranging from endoplasmic reticulum, to tonoplast. In the case of PDV, there is still no evidence of natural resistance sources in the host plants susceptible to virus infection. Apparently, PDV has a great ability to overcome the natural defense responses in a wide spectrum of plant hosts. The first manifestations of PDV infection are specific cell membrane alterations, and the formation of replicase complexes that support PDV RNA replication inside the spherules. During each stage of its life cycle, the virus uses cell components to replicate and to spread in whole plants, within the largely suppressed cellular immunity environment. This work presents the above stages of the PDV life cycle in the context of current knowledge about other *Bromoviridae* members.

## 1. Introduction

*Prune dwarf virus* is a member of one of the six genera in the family *Bromoviridae*. Genus *Ilarvirus* includes 19 species. Historically, this genus was divided into four subgroups according to serological characteristics [1] (Table 1), or six groups according to movement protein (MP) and coat protein (CP) coding sequences [2,3,4]. In 2016, in the genus *Ilarvirus*, new systematics were presented by International Committee on Taxonomy of Viruses (ICTV) , and now this group of viruses includes *American plum line pattern virus* (APLPV), *Apple mosaic virus* (ApMV), *Asparagus virus 2* (AV-2), *Blackberry chlorotic ringspot virus* (BCRV), *Blueberry shock virus* (BlShV), *Citrus leaf rugose virus* (CiLRV), *Citrus variegation virus* (CCV), *Elm mottle virus* (EMoV), *Fragaria chiloensis latent virus* (FCILV), *Humulus japonicus latent virus* (HJLV), *Lilac leaf chlorosis virus* (LLCV), *Lilac ring mottle virus* (LiRMoV), *Parietaria mottle virus* (PMoV), *Prune dwarf virus* (PDV), *Prunus necrotic ringspot virus* (PNRSV), *Spinach latent virus* (SpLV), *Strawberry necrotic shock virus* (SNSV), *Tobacco streak virus* (TSV), *Tulare apple mosaic virus* (TaMV) [5]. Similarly to PDV, viruses in this genus are transferred through seeds, pollen grains, and during vegetative propagation [6]. The generic name refers to the characteristic icosahedral symmetry of virus particles. Icosahedral virions of *Ilavirus* class T = 3 usually consist of 180 molecules of coat protein and encapsidate (+)ssRNA [7,8]. Icosahedral particles are also typical of the remaining genera: *Anulavirus*, *Bromovirus*, and *Cucumovirus*. Certain virus species in the genus *Ilavirus*, including PDV, form two types of particles at the same time: icosahedral and bacilliform [9,10]. The icosahedral (spherical) particles of PDV have a diameter ranging from 26 to 38 nm [6], whereas the bacilliform PDV have the length from 30 to 85 nm and the diameter from 18 to 26 nm. Bacilliform particles are characteristic for genera: *Alfamovirus*, *Ilarvirus*, and *Oleavirus.*

The genomes in all *Bromoviridae* members, including PDV, consist of three single-stranded (+)RNA (ssRNA) components, identified as RNA1, RNA2, and RNA3. Each of the (+)ssRNA particles possesses a cap structure at the 5′-end and a 3′-untranslated region (3′UTR), that forms a tRNA-like structure (TLS) [11]. Furthermore, during virus replication, a subgenomic RNA4 (sgRNA4A) is transcribed, and is responsible for translation of coat protein (CP). Also, in the genus *Cucumovirus*, and in the majority of *Ilarviruses*, an additional open reading frame (ORF2b) has been identified within RNA2, that is expressed from an additional subgenomic sgRNA4A; sgRNA4A encodes a suppressor of RNA interference (RNAi) [12]. Another characteristic of *Bromoviridae* is the occurrence of high concentrations of CP in the infected cells, but rather low amounts of non-structural proteins. The knowledge about replication cycle of PDV is still poorly understood, and limited mostly to the aspects of the structure and RNA genome organization.

## 2. Genome Organization of *Prune Dwarf Virus* (PDV)

The genome of PDV is divided into three single-stranded positive RNA segments RNA1 (1.3 × 10^−6^ Da), RNA2 (0.95 × 10^−6^ Da) [13,14], and RNA3 (0.76 × 10^−6^ Da) [15,16,17]. Each RNA component is individually packed into viral capsids [10,13].

**Figure 1:**
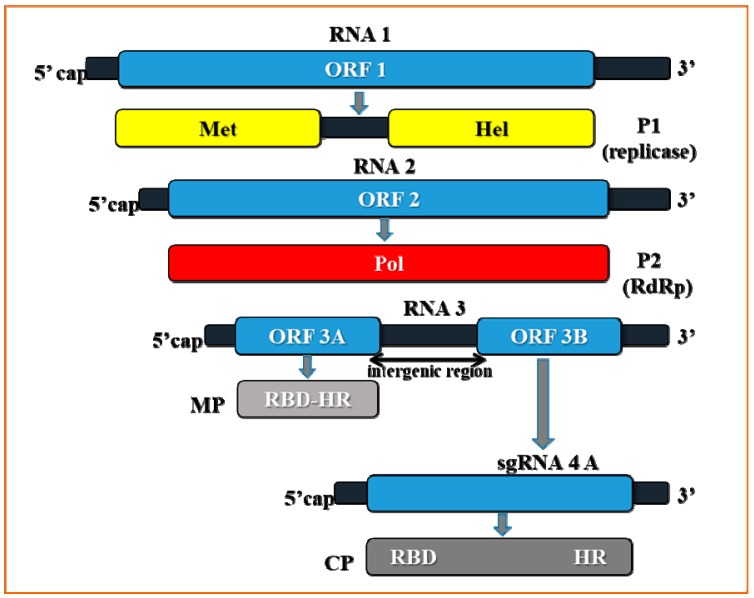
Genome structure of *Prune dwarf virus* (PDV). Scheme presenting the individual open reading frames (dark blue) and proteins (different colors) encoded by PDV RNAs. Encoded proteins: P1 with methyltransferase and helicase domains (yellow), P2—polymerase (red), MP—movement protein (grey), CP—coat protein (green). ORF—open reading frame, RBD—RNA binding domain, HR—hydrophobic region. Scheme of genome prepared on the basis of the data in Bujarski et al. [1].

The monocistronic PDV-RNA1 was first sequenced by Ramptish and Estwell [18] from the Canadian PDV isolate which originated from *Prunus avium* trees of the cultivar Salomo. RNA1 had a length of 3374 nucleotides, and contained single open reading frame (ORF) identified as ORF1, comprising 3168 nucleotides and coding for a non-structural protein P1 (putative replicase component) (Figure 1).

Similarly to RNA1, PDV-RNA2 is a monocistronic molecule carrying ORF 2 (Figure 1). Determined by Scott et al. [19], the full nucleotide sequence of RNA2 amounted to 2593 nucleotides, whereas ORF2 had 2367 nucleotides and coded for a non-structural protein P2, the RNA-dependent RNA polymerase (RdRp) component of the replicase [9]. This feature is in contrast to the structure of RNA2 in other *Ilarviruses*, as most of them carry the additional ORF 2b [7] (in sgRNA4A) well described for *Cucumber mosaic virus* (CMV). Contrary to RNA1 and RNA2, PDV-RNA3 is a dicistronic molecule (Figure 1). Determined by Bachman et al. [20], the full-length sequence of RNA3 of the American PDV-137 isolate consisted of 2129 nucleotides. The two RNA3 ORFs, ORF3a and ORF3b (sometimes identified as MP-ORF and the coat protein (CP)-ORF, respectively) [12] are separated by a 72 nucleotide intergenic region. Little is known about the role and composition of the intergenic region of PDV. The 5′ side ORF3a consists of 882 nucleotides and codes for movement protein MP. The 3′ side ORF 3b consists of 657 nts and codes for CP [21]. RNA3 is a well-studied entity, being most frequently sequenced and phylogenetically compared among different *Bromoviridae*. An analysis of the RNA3 sequences of two Polish isolates (PDV-D1, PDV-D2) and one German isolate (PDV-15/28) demonstrated that the number of nucleotides in RNA3 was 2129, similar to those in PDV-137. The homology of the RNA3 nucleotide sequences and the MP and CP amino acid sequences among PDV-D1, PDV-D2, and PDV-15/28 isolates are 96.9%, 93.8%, and 98.6%, respectively [22]. Also, Vaskova et al. [23] determined the homology of CP-ORFs in 11 PDV isolates from Czech Republic (five isolates from cherry trees, two isolates from peach trees and four from prune trees) to the amount over 88%.

Ulubas-Serce et al. [24] carried out full comparative analysis of the CP nucleotide sequences among PDV isolates obtained from almond trees (nine isolates from Portugal), cherry (20 isolates from Turkey, Germany, Canada, USA, Hungary, and Poland), prune (four isolates from Russia and Germany), peach (two isolates from Germany), apricot (one isolate from Turkey) and two isolates from unknown sources. The nucleotide sequence homology ranged from 87 to 99%, whereas at the amino acid level (218 amino acids), the homology ranged from 84–99%. Based on these results, the isolates were divided into four groups: cherry 1, cherry 2, almond, and mixed. The homology for mixed group ranged at 92–99%, cherry 1 at 95–99%, cherry 2 at 89–98%, and the almond group at the level of 86–95%.

Another study conducted by Bachman et al. [20], Ramptish and Estewell [18], and Scott et al. [19], indicated that major differences between PDV isolates occurred within the RNA3 sequences, which enabled differentiation of the particular phylogenetic groups. However, the RNA3 sequences were also strongly conservative, indicating key roles of MP and CP at early stages of infection, especially during virus transport and virus interaction with the cell elements [7].

## 3. The Crucial Functions of Proteins Coded by PDV RNA

**Figure 2:**
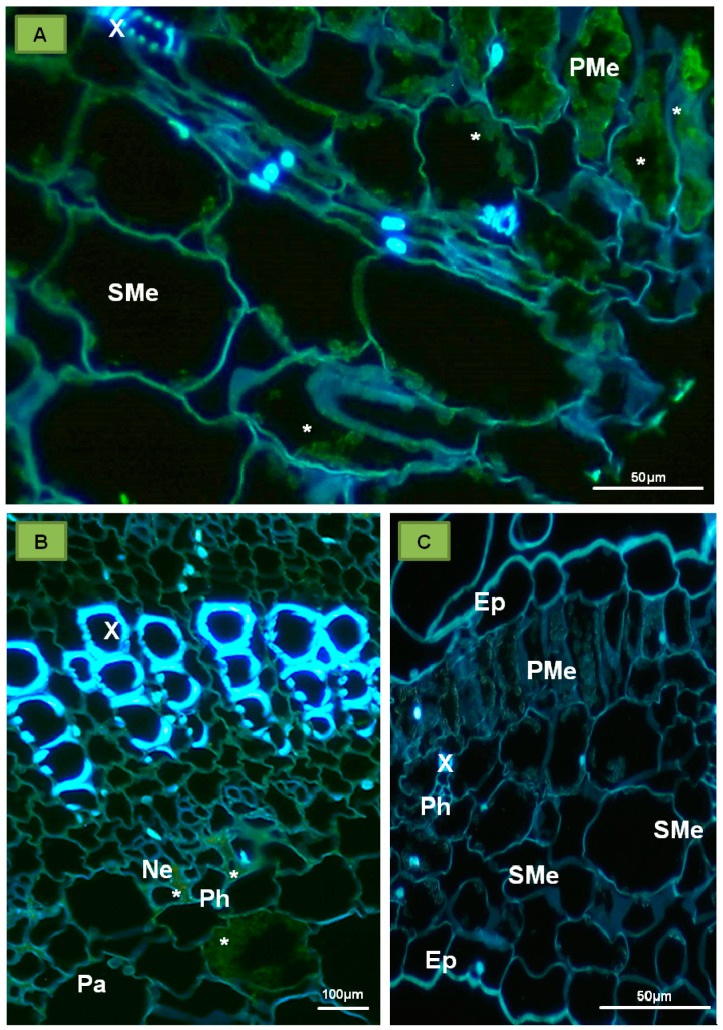
Immunofluorescent localization of P1 protein (replicase) in the tobacco leaf tissue of *Samsun* variety. (**A**) Immunofluorescent visualization of the epitopes of P1 protein (green, marked with *) in palisade and spongy mesophyll cells (cross-section of tobacco leaf blade); (**B**) Epitopes of P1 protein (*) visible in parenchyma and necrotic altered phloem; (**C**) Cross section of tobacco leaf 15 days after inoculation with buffer. No locations of P1 protein epitopes. Abbreviations: Ep—epidermis, PMe—palisade mesophyll, SMe—spongy mesophyll, Pa—parenchyma, X—tracheal element, Ph—phloem, Ne—necrosis. Kozieł et al. [32] modified.

**P1 protein**—this protein is encoded by PDV-RNA1 (Figure 1), and contains 1055 amino acids (molecular weight 110 kDa) [25]. Sometimes referred to as “putative replicase”, it is an enzymatic protein made of two domains and engaged in the viral RNA replication process. The N-terminal domain consists of 340 amino acids, and has a methyltransferase domain (MET). MET is responsible for attaching the 5′-terminal cap that protects the viral RNA against degradation [26]. The C-proximal domain contains 259 amino acids, and it likely has UvrD/REP helicase (HEL) activity, capable of unwinding the RNA strands during replication [18,26,27]. In addition, P1 is engaged in anchoring of the viral RNA, possibly inside a separate vesicle-like membranous structure, within which the replication complex is assembled [7,28,29]. Among the members of *Bromoviridae*, P1 can be linked to different cellular structures. For instance, P1 of BMV locates to ER [25], whereas for *Alfalfa mosaic virus* (AMV) and *Cucumber mosaic virus* (CMV), it targets the tonoplast [30,31]. Kozieł et al. [32] observed PDV-P1 epitope in both palisade and spongy parenchyma cells of tobacco (Figure 2A), as well as in necrotic phloem cells (Figure 2B). The epitope was not spotted in mock inoculated plants (Figure 2C) [32].

**Figure 3:**
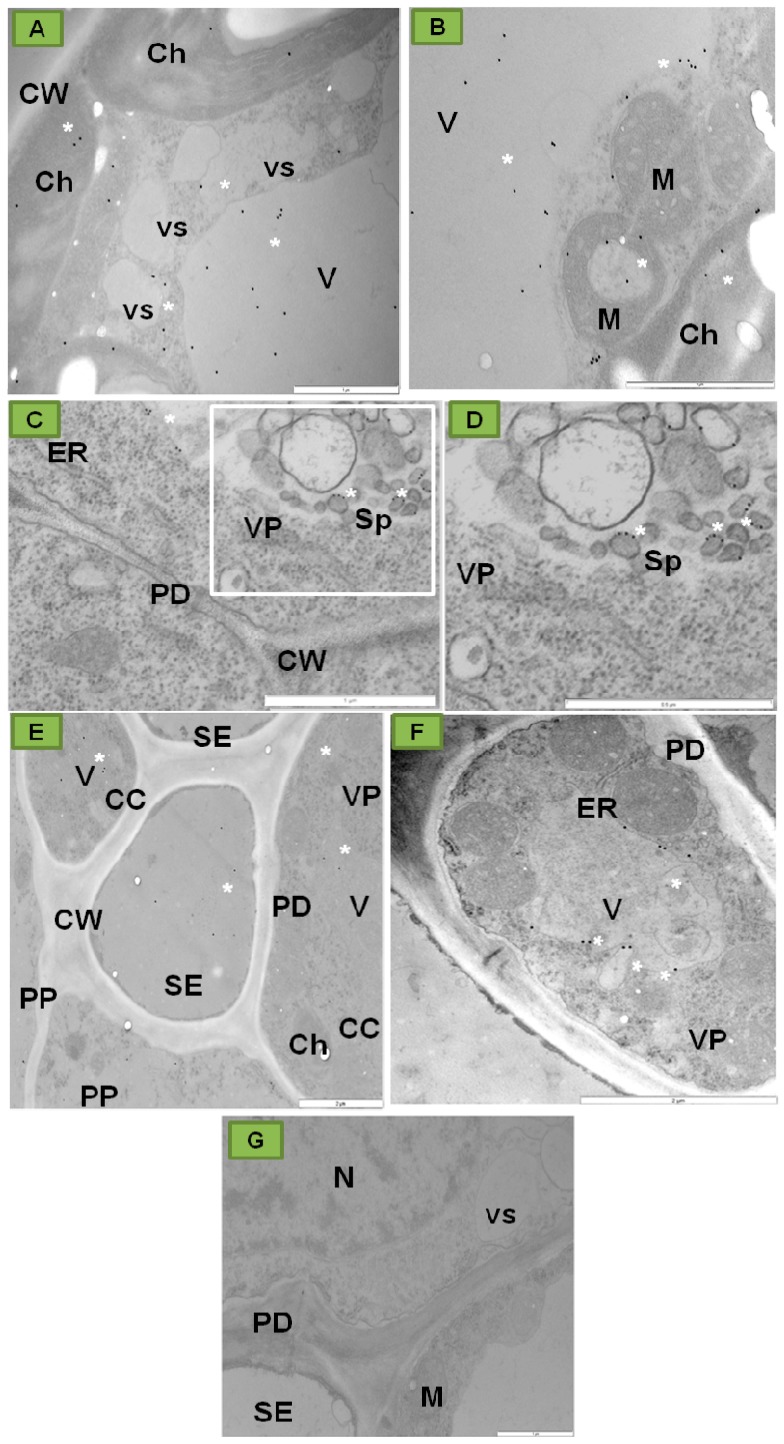
Immunogold localization of P1 protein (replicase) in mesophyll and phloem cells of tobacco leaf of *Samsun* variety 15 days after inoculation with PDV. (**A**) Colloidal gold particles associated with P1 epitope (*) in vacuoles, vesicles and chloroplasts of palisade parenchyma cell; Bar 1µm (**B**) Gold particles in palisade parenchyma cell (*) in chloroplast, vacuole, and in vicinity of mitochondria with electron-translucent area; Bar 1µm (**C**) Gold particles (*) in parenchyma cell tonoplast and in membranes of spherules. The white framed area is enlarged in (**D**); Bar 1µm (**D**) Enlarged fragment with spherules in the white frame from (**C**); Bar 0,5 µm (**E**) Epitopes of P1 protein (*) in vacuoles of phloem parenchyma and companion cells, and inside sieve tubes. Viral particles in companion cell; Bar 2µm (**F**) Colloidal gold particles (*) in companion cell vacuoles; Bar 2 µm (**G**) Control tobacco plant (mock-inoculated) phloem without of P1 localization Bar 1µm. Abbreviations: CW—cell wall, Ch—chloroplast, ER—endoplasmic reticulum, V—vacuole, vs—vesicle, M—mitochondrion, Sp—spherule, PD—plasmodesmata, SE—sieve tube, CC—companion cell, PP—phloem parenchyma, VP—viral particles, N—nucleus. Kozieł et al. [32] modified.

Moreover, PDV P1 localized strongly inside tonoplast (Figure 3A,B) and in spherules (Figure 3C,D) of different parenchyma cells or inside sieve tubes (Figure 3E) and vacuoles of companion cells (Figure 3F) [32]. In contrast to infected tobacco, no presence of P1 protein epitopes was observed in control plants (Figure 3G).

**P2 protein**—this protein contains 788 amino acids with molecular weight 89 kDa [21]. P2 is likely an RNA-dependent RNA polymerase (RdRp) (Figure 1) enzyme that, together with P1, forms the RNA replication complex [33]. Translation of P2 from RNA2 is direct, and the protein exhibits strong homology within the C-side throughout the entire *Bromoviridae* family [1].

**Figure 4:**
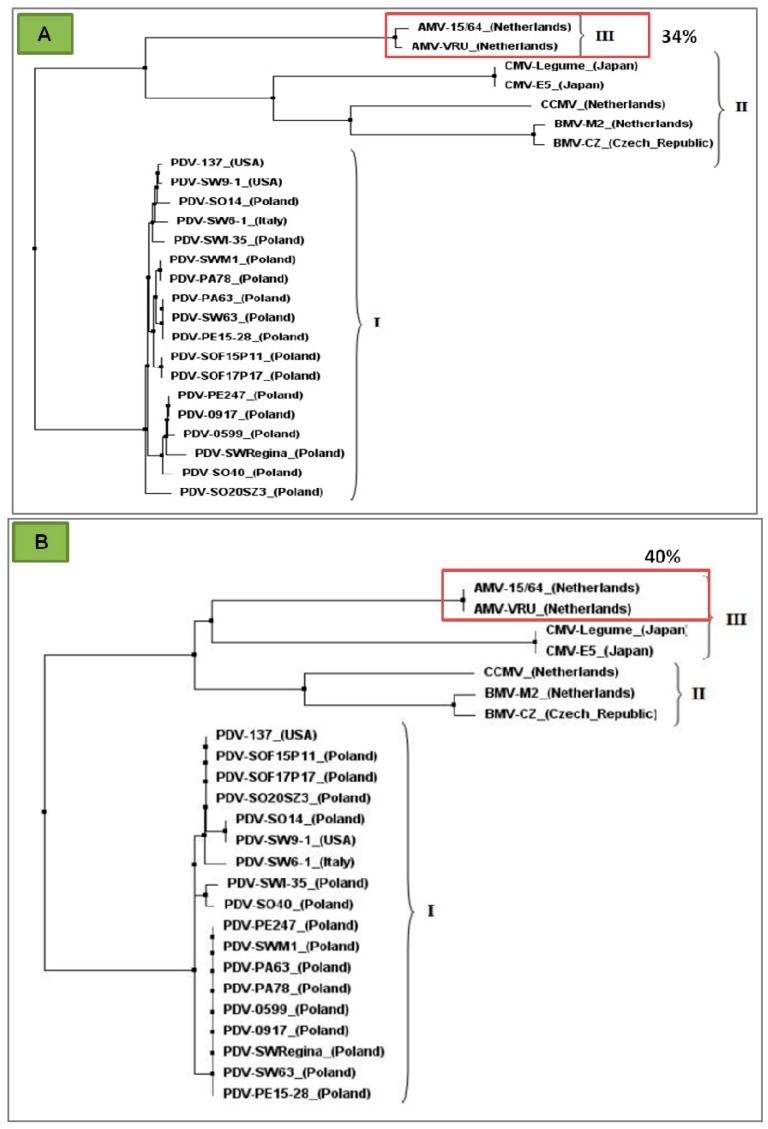
Phylogenetic comparison of amino acid sequences of the MP and RNA binding domain of PDV with several members of *Bromoviridae* family: (**A**) MP sequences for the studied groups of virus isolates. The highest similarity (maximum likelihood) between PDV and AMV marked in red frame (34%) (modified [36]); (**B**) RNA binding domain of the MPs. The highest similarity (maximum likelihood) between PDV and AMV marked within the red frame (about level 40%), Kozieł et al. [36] modified. On (**A**)and (**B**): I- group of analyzed PDV isolates sequences of movement protein, II- group of analyzed *Brome mosaic virus* (BMV) and *Cucumber mosaic virus* (CMV) isolates sequences of movement protein, III- group of analyzed AMV and *Cowpea chlorotic mottle virus* (CCMV) isolates sequences of movement protein.

**MP (movement protein)**—a transport protein with molecular weight of 32 kDa, contains 293 amino acids, and is subjected to direct translation from RNA3 (Figure 1). MP belongs to the superfamily of viral proteins “30K” [34], responsible for local transport of viruses [35]. Proteins of this family are characterized by a strongly conservative RNA-binding domain (RBD). RBD can be found in the genus *Ilarvirus* between residues 56–85, and it possesses a characteristic hydrophobic region, HR. RBD not only enables binding of the viral RNA, but it probably also supports its transport. It has not been determined whether in the genus *Ilarvirus* MP stimulates the formation of tubular structures in infected plant cells. However, it has been found that MP from PNRSV is capable of transporting AMV particles [7]. The immunolabeling analyses conducted with protoplasts from plant cells infected with AMV indicated that MP, similarly to CP, localizes itself in the formed tubular structures. Kasteel et al. [35] demonstrated that the MP proteins can support viral transport through tubular structures. Kozieł et al. [36] showed that the RNA binding domains (RBD) of PDV–MP were most similar to AMV–MP sequences. The similarity reached 34% and 40%, respectively, for the entire MP sequence and RBD (Figure 4A,B).

**CP (*coat protein*)**—coat protein has molecular weight of 23–24 kDa, consists of 218 amino acids, and its translation occurs via subgenomic (sg) RNA4 [21] (Figure 1). CP is a structural protein, but it also functions in genome activation to initiate infection [7,8]. The N-terminal CP fragment in *Ilarvirus* is rich in arginine (R) and/or lysine (K) residues that are responsible for binding to the 3′UTR hairpin structure of viral RNA [37,38]. This binding transforms the hairpin into a pseudoknot structure, similar to that of TLS [39]. The pseudoknot structure is stabilized and maintained with Mg^2+^ [7], which reduces binding of subsequent CP molecules, but stimulates RNA binding to the replication complex. In the case of *Prunus necrotic ring spot virus* (PNRSV), a virus closely related to PDV, a specialized arginine-rich domain of CP displays the affinity to 3′UTR in both RNA3 and sgRNA4A. The domain is located between 25 and 50 N-terminal amino acids, probably in all *Ilaviruses* [40,41]. In two viruses, *Apple mosaic virus* (ApMV) and PNRSV, a zinc finger motif was identified within CP, which probably increases the affinity for RNA binding.

The C-terminal region plays key role in CP dimerization. Bol [28] suggests that CP, apart from genome activation or from virion formation, is also engaged in other processes, including the asymmetric (+)/(−) strand RNA synthesis, translation of viral RNA, and both intercellular and systemic transport of *Ilaviruses*.

**Figure 5:**
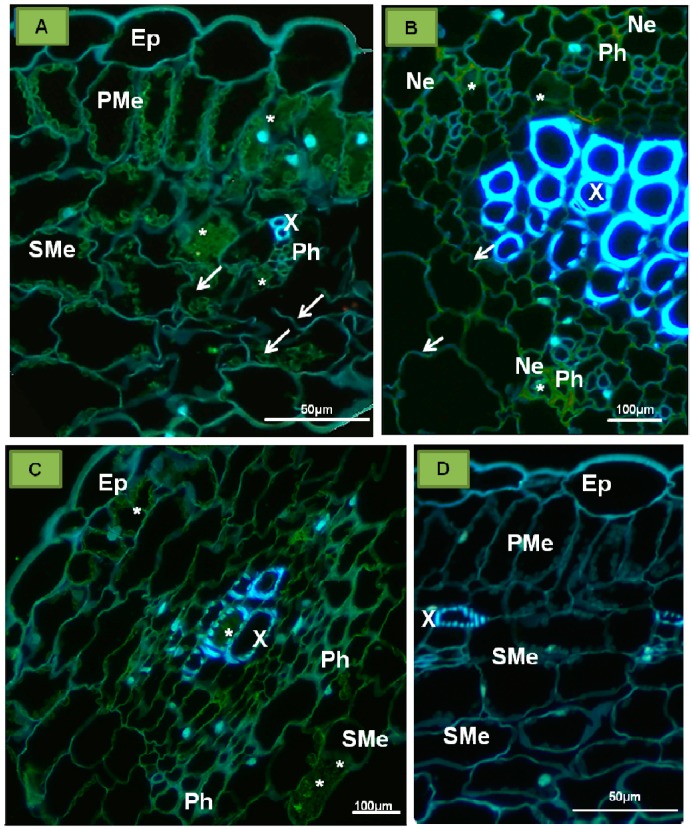
Immunofluorescent localization of coat protein in tobacco leaf of *Samsun* variety 15 days after inoculation with PDV. (**A**) The epitopes of coat protein (*) in phloem cells, palisade, and spongy mesophyll. Disintegration of spongy mesophyll cells observed (arrow); (**B**) The epitopes of coat protein (*) in necrotic phloem cells. Visible cell wall invagination of parenchyma cells (arrow); (**C**) The epitopes of coat protein (*) in the spongy mesophyll cell and tracheal element; (**D**) Fragments of leaf blades inoculated only with buffer after 15 days. No CP epitopes observed. Abbreviations: Ep—epidermis, PMe—palisade mesophyll, SMe—spongy mesophyll, X—tracheal element of xylem, Ph—phloem, Ne—necrosis. Kozieł et al. [32] modified.

Moreover, Neelman and Bol [42] postulated for tobacco protoplasts infected with AMV (member of *Bromoviridae* family like PDV) that collecting and individual packaging of RNA particles to the capsids are influenced by the spatial conformation of CP. Coat protein has always been considered to be responsible for supporting the replication process and later encapsidation, via in *trans* effects on RNA 1 and RNA2 but in *cis*—on RNA3. Rao [43] also developed a model of sequential RNA3 encapsidation process for with particles carrying the subgenomic RNA. In this model, encapsidation consists of three subsequent stages. In stage I, CP subunits recognize a two-part signal consisting of nucleotides at the 3′ TLS end of RNA3, referred to as the nucleating element (NE), and of the ORF coding MP. After the signal recognition, the RNA3 molecule is packed to the capsid. Then, the arginine residues from RBD CP, located on the surface of the virion, bind sgRNA4A (stage II), which is packed together with RNA3 (stage III) [43]. At this moment, however, a similar mechanism of encapsidation in the case of PDV is uncertain. The presence of PDV CP epitopes has been demonstrated in palisade parenchyma (Figure 5A), necrotically altered phloem (Figure 5B,C), and even in xylem treachery elements (Figure 5C) [32]. In the mock-inoculated plants, no CPs were observed (Figure 5D). Similar results were presented by Silva et al. [44] by using the in situ reverse transcription-polymerase chain reaction assay on the almond leaves. These authors [44] have demonstrated the presence of virus (by genome fragment which encoded CP) in both mesophyll and vascular tissues in young leaves. In the case of almond plants, this technique has also enabled the recognition of PDV particles inside the generative organs [44].

**Figure 6:**
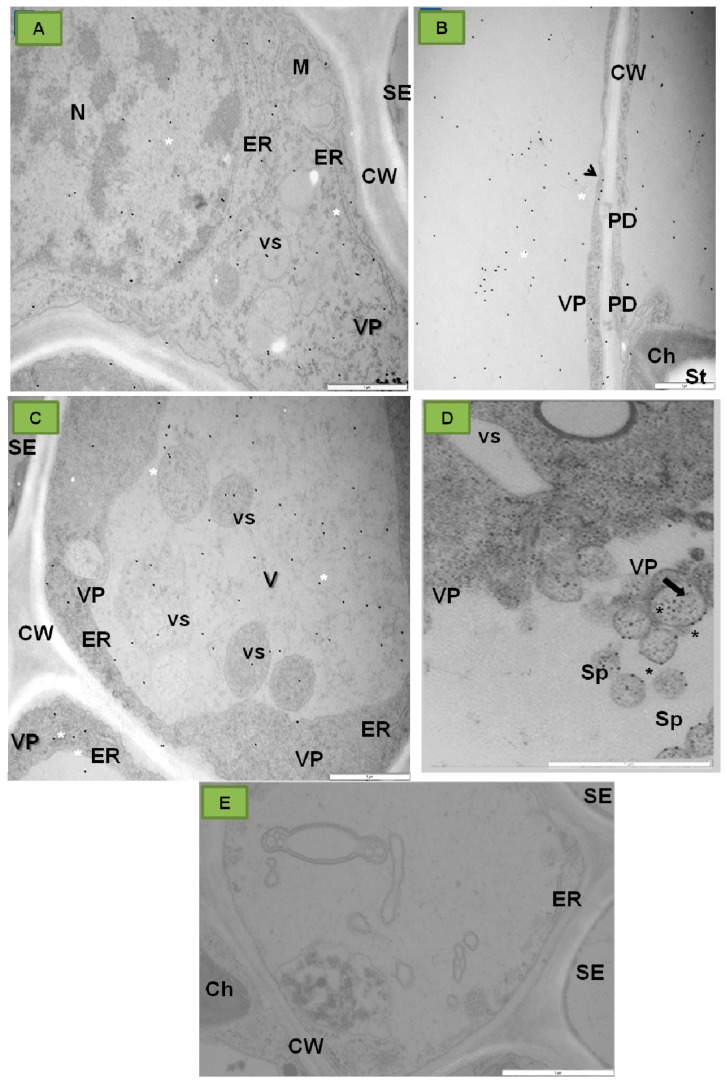
Immunogold localization of PDV coat protein (CP) in the tissues of the tobacco leaf blade, *Samsun* variety, 15 days post inoculation with PDV. (**A**) Labeling is observed in nucleus (N) and on the endoplasmic reticulum (ER) surface (*); (**B**) Colloidal gold particles (*) in vacuole of mesophyll cell. Labeling observed in cytoplasm (arrow) and by plasmodesmata; (**C**) Colloidal gold particles (*) in the companion cell protoplast. Labeling is observed both in vesicles and in the vacuole; (**D**) Colloidal gold particles (*) in the spherules’ membranes in a parenchyma cell; (**E**) Control tobacco plant phloem without of CP localization. Abbreviations: CW—cell wall, Ch—chloroplast, M—mitochondrion, ER—endoplasmic reticulum, VP—viral particles, vs—vesicle, SE—sieve tube, Sp—spherule, PD−plasmodesmata; St—starch−, Bars 1 µm.Kozieł et al. [32] modified.

Moreover, these authors observed PDV CP inside endoplasmic reticulum (ER) (Figure 6A), near plasmodesmata (Figure 6B), in vacuole, and in tonoplasts (Figure 6C) of tobacco cells. The presence of PDV CP in the spherules has also been observed (Figure 6D). In the case of mock inoculated tobacco plants, the PDV CP epitope was absent (Figure 6E).

Phylogenetic analyses of sequences of PDV CP showed a high level of diversity among the diverse isolates from remote parts of the world. Kalinowska et al. [15] showed a diversity of 86–100% at nucleotide level, and 79–100% at the amino acid level. The ratio of non-synonymous to synonymous polymorphic sites indicated that purifying selection dominated in the case of PDV. However, based on the analysis of six codons, they also showed that the codons were under strong positive selection, including a codon involved in the RNA-binding activity [15]. Among Turkey PDV isolates, Öztürk et al. [45] demonstrated 84–99% to 81–100% nucleotide and amino acid CP sequence identity, respectively, after comparing PDV isolates from Isparta and from other parts of the world. However, despite differences in serological and biological properties among PDV isolates, a molecular characterization of viral coat protein sequences did not confirm a correlation between amino acid composition and host range and/or the origin of viral isolates [23]. It is most likely that the differences between isolates emerged frequently in different parts of the world [15]. Such wide diversity confirms a great ability of PDV to overcome the natural defense responses in a wide spectrum of plant hosts.

## 4. PDV Infection Cycle Based on the *Bromoviridae* Family Model

The initial stage of viral infection is mechanical or biological discontinuity of the plant cell wall, to enable virus penetration through cellular membrane. So far, no specific receptors have been identified for penetration of plant viruses [12,46]. However, the electron microscope (EM) studies carried out on isolated protoplasts have shown that *Bromoviridae* could be absorbed by cellular membrane via pinocytosis [7,47]. In the case of PDV, the virus may be transmitted mechanically among the plants [13]. Greber et al. [48] showed also that PDV under laboratory conditions (but not in the field) may be transmitted to cucumber plants by thrips (*Frankliniella occidentalis*), with the transmission level nearly 20%.

After passing the cellular membrane, certain *Bromoviruses* induce proliferation of endoplasmic reticulum (ER) in the form of spherules and/or the formation of small vacuoles from the external nuclear membrane, which constitute the site of initial viral infection. In the case of PDV, the presence of CP epitopes inside the tobacco spherules has been observed near vacuole rather than ER (Figure 6C,D) [32], suggesting the PDV-induced proliferation in the tonoplast membrane.

**Figure 7:**
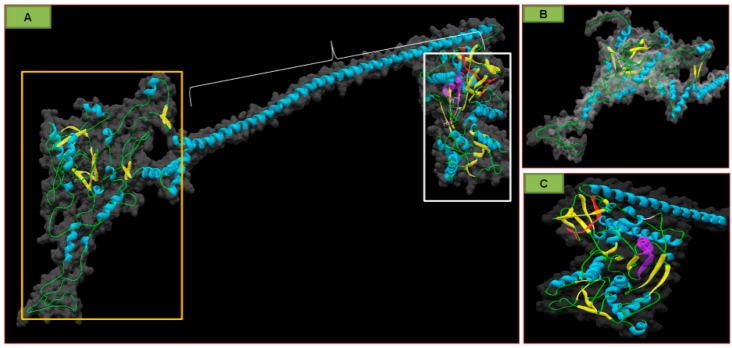
Three-dimensional (3D) model structure of the PDV replicase (P1) protein, considering the surface of both N- and C-terminal domain, and the transmembrane domain (modified Kozieł et al., 2017). (**A**) The colors show the particular elements of the secondary structure, as follows. Green indicates the fragments of straight polypeptide chain, blue—α-helical fragments, yellow—β-card fragments; orange—the frame of methyltransferase domain, white—the frame of helicase domain. The central buckle depicts the helical region between both domains. The protein framework of the 3D structure is marked in gray; (**B**) Magnification of the methyltransferase domain from Figure 6a. Gray indicates protein surface; (**C**) Magnification of the helicase domain (from Figure 6A) rotated 90 degrees vis-à-vis the vertical axis, with the region used for immunolocalization of P1 marked in red. Purple indicates the predicted transmembrane domain. The area of the general framework of the helicase C-terminal domain is represented by the gray color. Kozieł et al. [32], modified.

Each viral particle encapsidates a single, translation-competent (+)ssRNA, and proteins 1a and 2a are first to be translated. The translation process is stimulated by the 3′TLS region that interacts with the cap at the 5′ side, and secures the circularization of the RNA template [11,49]. Pallas et al. [7] postulate that, similarly to AMV, an efficient translation in *Ilarviruses* (for example PDV) requires the presence of CP molecule at both the 3′ and 5′ sides to maintain a correct RNA conformation. Moreover, the translated replicase proteins are stored in different cellular compartments, depending upon a particular virus. BMV stores them on the ER membrane, AMV, and CMV in the tonoplast or in the vicinity of the vacuole [46,50,51]. Kozieł et al. [32] suggested that PDV P1 protein carries a transmembrane domain between 848 and 869 amino acids (the helicase portion, marked purple on Figure 7A,C) [32]. The rest of this protein comprises a methyltransferase domain (Figure 7A,B).

**Figure 8:**
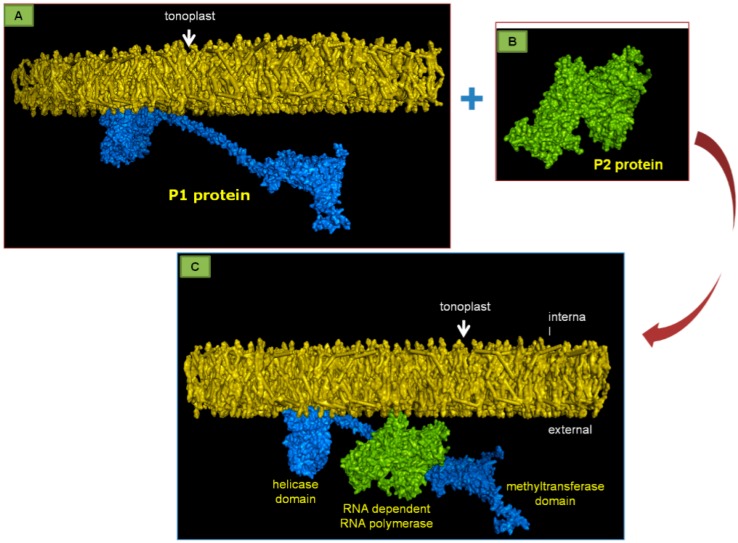
(**A**) 3D visualization of a model of the PDV replication complex that assembles during the first steps of viral RNA replication; (**A**) P1 protein (of the replicase complex), marked by blue color, as it anchors to the tonoplast membrane; (**B**) P2 protein (RNA dependent RNA polymerase) 3D surface structure, as marked by green color. (**C**) Fully assembled replication complex, consists of P1 protein (in blue) attached to tonoplast membrane and P2 protein (in green), that is attached to P1 between its both domains. This computer-generated model was generated by using THMM program, in ΔG Prediction Server 1.0, and in AIDA server. To display the results of these calculations, the CELLmicrocosmos Membrane Editor was utilized.

The immunogold labeling of CP and P1 PDV demonstrates that, as for AMV, its replication is primarily associated with the tonoplast [31]. The replication complex also contains the RNA-dependent RNA polymerase (P2 protein) [26]. PDV P1 is firstly attached to the tonoplast membrane (Figure 8A) and then P2 (Figure 8B) is connected (Figure 8C), making a functional replication complex. PDV P1 brings and anchors the viral RNA to the tonoplast membrane [32]. In addition, P1 supports the replication process, whereas P2 is directly responsible for RNA synthesis. The replication complex generates both the (+) strand (the encapsidated strand) and the antisense (−) strand that is used as template for the (+) strand synthesis [8].

With *Bromoviridae*, the two RNA strands are synthesized asymmetrically; one (−) RNA molecule accounts for approximately 100 (+) RNA molecules [52]. Bol [28] suggested that asymmetrical replication is also characteristic for *Ilarviruses* like PDV. It seems that CP acts as the factor responsible for asymmetrical synthesis of RNA strands [28]. Olsthorn et al. [53] proposed a model of conformational switch affecting the accumulation of sense and antisense strands in AMV. Similarities between AMV and PDV suggest a similar mechanism for both viruses. In this model, CP binds to the 3′UTR, and prevents formation of the pseudoknot structure, and thus prevents RdRp from initiating (−) strand synthesis, promoting the asymmetrical accumulation of (+) strands. The (−) strand promoter includes an AUG loop in 3′UTR TLS, whereas for the plus strands, this is located at the 3′ end of the antisense strand [54]. On the other hand, the 3′UTR of CMV directly interacts with the eukaryotic transcriptional factors eIF4E and eIF4G, and regulates the transcription process [55,56]. The newly-synthesized viral RNA is then interacting with MP, and gets transported to regions where virions are assembled.

## 5. The Specificity of Cell-to-Cell Transport in Different Plant Viruses as Compared to PDV

Plant viruses are specialized pathogens able to move in plants by using cellular structures of the host [43,57,58]. Infection begins in the epidermal cells; the virus first moves from one cell to another to the mesophyll, bundle sheath, and parenchyma, then to phloem via the accompanying cells or xylem [43,57,58,59,60]. The cell-to-cell transport enables penetration and spread among the cells, and can be described as a process consisting of three main stages: (i) transfer of newly synthesized genomes/virions from the replication/assembly site to the intracellular transport system [57]; (ii) direct, facilitated transport of genome/virions to reach the plasmodesmata [35]; and (iii) transport to new cells via plasmodesmata [11,61,62].

The long-distance transport of viruses is closely related to the transport that occurs from the phloem parenchyma cells or accompanying cells to the interior parts of the sieve elements in the phloem, where the virus moves rapidly (several centimeters per hour). Then, the virus is actively transported together with assimilates inside the sieve elements [63,64,65,66]. Less frequent systemic transport involves the tracheal elements of xylem. In fact, the virus uses the preexisting network of symplastic connections for systemic infection of the plant host [67]. There are two major types of intercellular transport among various plant viruses, and both types can be found in different *Bromoviridae*.

**Figure 9:**
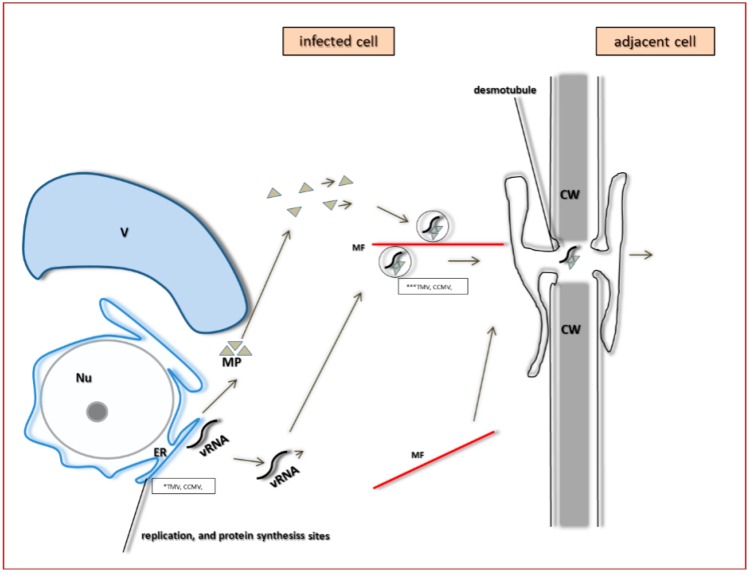
Model of the pathogen life cycle, from replication to cell-to-cell transport, by compiling both TMV and CCMV as the examples of viral RNA (vRNA) replicated at the endoplasmic reticulum (ER), and then transported as a complex with movement protein (MP) along actin microfilaments (MF) to the plasmodesmata (PD) region. Simultaneously, MP is also transported alongside microtubules to PD. MP modifies the size exclusion limit (SEL) of PD. Frame with *TMV, CCMV replication sites of vRNA. Frame with ***TMV, CCMV complexes of vRNA and MP, transported with help of MF.

The first group includes two subtypes [68]. One subtype, described well for *Tobacco mosaic virus* (TMV) (*Virgaviridae*, *Tobamovirus*) does not require CP for transport (Figure 8). The virus is transported in a form of its genome (the non-virion form) [69]. Here, the transfer of the viral genome from the cytoplasmic replication site involves the existing intracellular transport systems [70]. The TMV MP forms complexes with the (+) single-stranded viral RNA (vRNA), where one MP monomer accounts for 4–7 nucleotides [71,72,73]. TMV and other viruses with similar MP proteins of molecular weight around 30 kDa and with a characteristic RNA binding domain, form the “30K” transport protein superfamily [34]. TMV–MP and TMV RNA form a non-undulating structure of the width around 1.5–2.0 nm, that is transported from the membranous replication site to plasmodesmata by using the microtubules and microfilaments of cellular cytoskeleton to direct the transport [73,74,75] (Figure 9). Studies demonstrated the co-location of MP with microtubules and subunits of actin filaments in protoplasts co-transported with the MP-GFP constructs. TMV–MP has also the potential to interact with tubulin and actin in vitro. Due to facilitated transport, microtubules support direct movement of large complexes, macromolecules, organelles, vesicles, and mRNA [62,76,77]. It is possible that the TMV–MP interaction may constitute an example of molecular mimicry, because MP contains motifs characteristic of tubulin [78]. Viruses with mutation in the tubulin motif did not bind their MPs to tubulin, and were characterized by decreased intercellular spreading rate. Boyko et al. [79,80] demonstrated the necessity of MP interaction with microtubules in order to secure the correct process of TMV transport. So far, two models of the transport of TMV–RNA–MP complex using microtubules have been proposed. The first model indicates the possibility for active transport of the complex, due to participation of kinesin. The second model predicts that the complex is transported thanks to the retraction folding/elongation of microtubules. Studies from recent years lean toward the second model [81,82]. Probably, during the early stages of infection, the TMV–MP co-locates with EB1 protein (end-binding protein **1**), that is bound to the plus end of microtubules, which then implies the possibility of MP elongation during transport. Application of herbicides that block microtubule polymerization also blocked the transport of viral complexes [82]. However, in late infection stages, TMV–MP likely remains immobile and bound to microtubules. The complex located near the plasmodesmata penetrates to ER, and via desmotubule, passes the boundary between cells. With the help of MP, TMV can efficiently utilize the cytoskeleton structures, and can protect the pathway of the viral complex to the neighboring cell [73]. The question still remains whether transport of such complexes requires myosin or kinesin, the motor proteins. TMV–MP also has the ability to anchor at ER membrane, enabling the transport from the membrane structures. N and C ends of MP are located on both sides of the cell membrane [83,84]. Thus, viral RNA is most likely transported as a ribonucleoprotein complex (vRNA–MP) (Figure 4). An analogous mechanism to that of TMV was demonstrated by Rao [85] for *Cowpea chlorotic mottle virus* (CCMV), the *Bromoviridae* representative. In this case the transport protein locates itself in plasmodesmata and causes considerable increase of the lower level of size exclusion limit, SEL. As a result, the plasmodesmata widened to the extent that the complex with the viral genome was able to penetrate through to the next cell [85,86].

**Figure 10:**
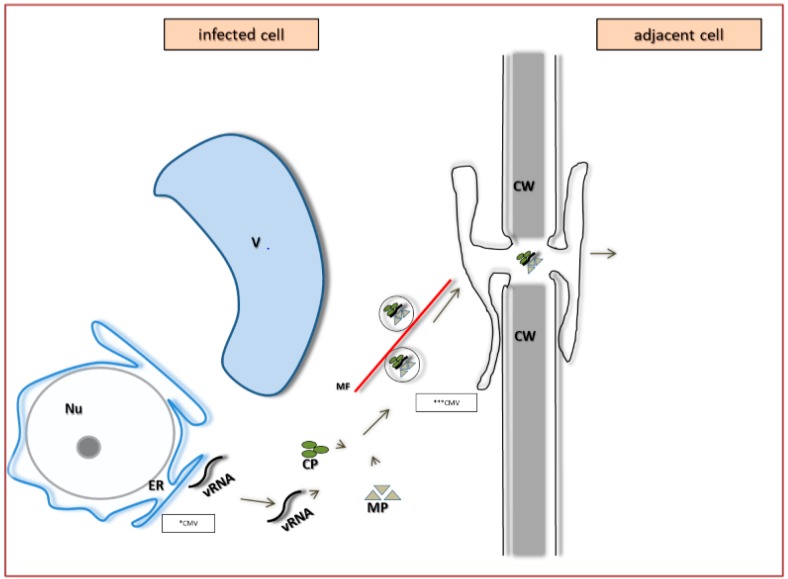
Model of a viral pathogen life cycle, from replication to cell-to-cell transport, based upon CMV characteristics. Viral RNA (vRNA) replication occurs at the endoplasmatic reticulum (ER) and the RNA is transported along actin microfilaments (MF) to plasmodesmata as a complex consisting of coat protein (CP) and movement protein (MP). MP modifies the size exclusion limit of plasmodesmata. Frame with *CMV-replication sites of vRNA. Frame with ***CMV vRNA, MP and CP complex transported with help of MF-route (mechanism) of cell-to-cell transport.

The second subtype depends on both CP and MP (Figure 10). It is characteristic for *Cucumber mosaic virus* (CMV) [87]. Canto and Palukaitis [88] demonstrated that CMV–MP induces formation of tubules in the infected protoplasts. Mutations of C-terminal amino acids of MP blocked tubulin synthesis, without limiting the cell-to-cell transport. Sztuba-Solińska and Bujarski [12] suggested that tubules support transport, but are not indispensable. Su et al. [89] demonstrated that MP binds near the ends of the actin microfilaments, which may indicate the participation of the actin cytoskeleton in the transport process. In the case of CMV the transported form is the ribonucleoprotein complex containing three components: vRNA, MP and CP. Correct CP and MP structures are of paramount importance for the complex formation and their mismatching does block the virus transport. Most likely, MP brings the viral RNA to the ribonucleoprotein complex. The vRNA–CP–MP complex is transported along actin filaments to reach the plasmodesmata (Figure 10), where MP probably stimulates the SEL increase [69].

The second group of movement mechanisms operates e.g., in *Cowpea mosaic virus* (CPMV, *Secoviridae*, *Comovirus*) or in two *Bromoviridae*: *Alfalfa mosaic virus* (AMV) and *Brome mosaic virus* (BMV) [90,91,92,93,94]. MPs of BMV and AMV induce formation of tubular structures on the surface of infected protoplasts [35,95]. Kaido et al. [96] established that for movement of BMV in tobacco cells, the MP–BMV needs to bind to the cytoplasmic protein *Nb*NACa1. This protein has a similar sequence to MP–BMV, and is probably involved in the translocation of newly formed virions.

**Figure 11:**
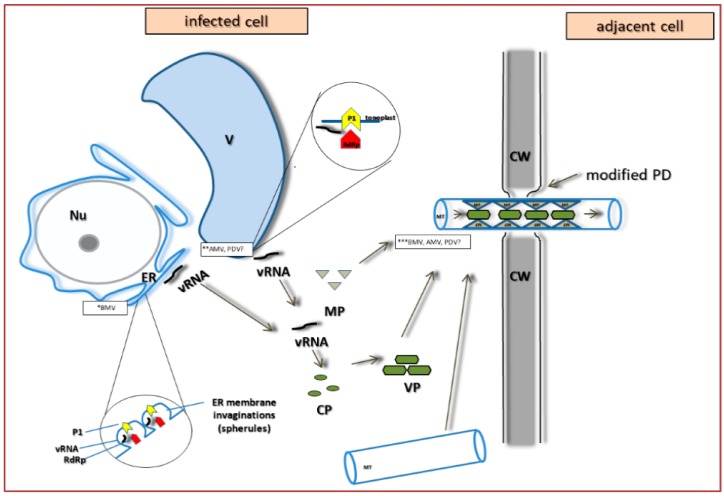
A model of PDV life cycle including replication and cell-to-cell transport, based upon research data for BMV and AMV. The BMV replication complex (P1 and P2) is assembled inside the spherules of the endoplasmic reticulum (ER) membranes. Viral RNA of BMV is encapsidated inside virion shells composed of CP molecules. In contrast, in the case of AMV and likely of PDV, the assembly of the replication complex and RNA replication are connected with the tonoplast membrane. As for BMV, the AMV and PDV RNAs are encapsidated with CP molecules. Thereafter, the assembled BMV or AMV (and likely PDV) virions are transported inside the microtubules (MT) that were modified with MP molecules. MP both change the PD size exclusion limit (SEL), but also destroy desmotubule structures inside PD. Frame with *BMV—replication sites of vRNA. Frame with **AMV, PDV replication sites of vRNA. Frame with ***BMV, AMV, PDV viral particles transported with help of MP an MT—in the case of PDV, probably route (mechanism) of cell-to-cell transport.

Mutations in the gene coding for *Nb*NACa1 limited both the location of MP in plasmodesmata and the BMV movement. Both N- and C-MP termini are responsible for the induction of cytoskeleton protein synthesis, as demonstrated with corresponding MP mutants that blocked the formation of tubules in protoplasts [96]. MP not only induces, but also penetrates to the interior of the tubular structures. Studies on MP from PNRSV, a virus closely related to PDV, demonstrated that the characteristic HR domain has strong affinity to cell membranes, and probably anchors the protein in the membrane [68]. Deletions in HR domain prevents PNRSV translocation through the cell membrane [83]. For this type of mechanism, the transported form is the virion, where CP binds to MP and both cover the interior parts of microtubules that are connected to plasmodesmata (Figure 11).

Kozieł et al. [36] used bioinformatics to analyze the amino acid sequences of MP in PDV and in other *Bromoviridae* members. The authors showed that the sequences of MP RBD (movement protein RNA binding domain) among PDV isolates were most similar to AMV, suggesting a similar mechanism of cell-to-cell transport, likely in the virion form. Van der Vossen et al. [94] demonstrated that the deletion of a considerable C-terminal portion of AMV CP prevented virion formation, but maintained the ability of cell-to-cell transfer. Sanchez-Navarro et al. [97] showed that the normal cell-to-cell transport of AMV, required a 44 amino acid C-terminal MP sequence. Moreover, the same authors have shown that the replacement of this 44 aa sequence with the corresponding region of MP from BMV, PNRSV, or CPMV supported the intercellular movement of AMV. Apparently, these proteins use a very similar mechanism, with a key role for the N-terminal amino acids. MP AMV is often located in ER, and has the capability to move between cells [98].

Apart from the local transport via plasmodesmata, an equally important issue is the fast long-distance movement to secure efficient viral infection within distant plant organs. Phylogenetic comparison of the amino acid sequences of the RBD region (residues 56–85) among PDV strains and with those in the *Bromoviridae* members of known transport mechanisms revealed similarities between AMV and PDV [36], suggesting a similar mechanism of transport. This suggestion supports the presence of PDV particles in cells and CP epitopes near plasmodesmata in infected tobacco [32].

Thus, PDV is most likely transported in a form of viral particles, not only via plasmodesmata but also over long distances.

## 6. Systemic Transport of PDV and Other *Bromoviridae*

Long-distance transport, also referred to as systemic, often requires the virus to move from epidermis or parenchyma cells to penetrate the phloem parenchyma cells, followed by movement to the accompanying cells and/or sieve tubes. Until recently, it was believed that phloem is the only tissue involved in systemic transport. Indeed, Garbaczewska et al. [64] demonstrated that tobacco rattle virus (TRV) utilizes phloem during the transport. In addition, however, TRV moved from external tissues (parenchyma and epidermis) towards xylem parenchyma, and then to the tracheal elements (xylem vessels). The virus particles were observed in the sieve tubes as well as in the vessels. Thus, systemic transport of TRV is linked to both vascular tissues. Regardless of the selected vascular tissue, the next stage always relies on the virus spreading to other plant organs, and then the penetration from the vascular bundles to the neighboring tissues [65,99]. Plasmodesmata link the epidermis, mesophyll cells, and the vascular system, including sieve elements [61].

There are two key points in the pathway to enter and to leave the sieve elements. Plasmodesmata connecting the sieve elements with the accompanying cells display a unique morphology, namely the occurrence of extensive branching at the side of the accompanying cells [62]. At the side of the sieve elements, the plasmodesmata form a pore that does not contain ER, despite the fact that ER cisterns occur inside the sieve elements. Analyzes of the SEL (size exclusion limit) suggest that plasmodesmata between the accompanying cells and sieve elements are gated by volume, differing from those in other plant cells. As a reaction to viral infection, the plant often blocks the sieve elements with callose to reduce viral systemic spread [8]. A study on the accumulation of viruses in the secondary nervation system suggests that selecting a route to the sieve elements always involves the elements of the phloem parenchyma [84]. In all cases, when the accompanying cells became infected, the phloem parenchyma cells were also infected.

Not all viruses can penetrate to the sieve elements through phloem parenchyma; the plasmodesmata that connect directly the phloem parenchyma to the sieve elements can constitute an alternative potential route to phloem [100,101]. Sieve elements are not able to synthesize proteins (lack of ribosomes). Pallas et al. [7] and Hipper et al. [84] indicate that regulation of systemic transport, including virus transport, to and from sieve elements, may require participation of both the viral and host factors. Assembled virions moved efficiently between cells, but were incapable of long-distance transport via phloem [101,102]. CP is definitely required for long-range transport of TMV, suggesting that in phloem, the virus is transported as viral particles. Similarly, *Bromoviridae* require unaltered CP to move systemically [62,84,103]. Fajardo et al. [62] showed that AMV requires an unaltered sequence of 44 amino acids at the C terminus of the MP to move systemically in tobacco. These amino acids probably interact with CP to support systemic transport; similar results were observed for BMV and CMV [104,105]. It is likely that the virions constitute the systemically-transported form of *Bromoviridae* (including PDV), regardless of the differences in the mechanisms of cell-to-cell transport. Virion formation/encapsidation is the last stage in the virus life cycle. CMV is transported between cells as a ribonucleoprotein, and CMV encapsidation takes place within the wall elements of the tube after penetrating to smaller sieve tubes [87]. As demonstrated by Requena et al. [106], CMV is further transported as virion particles. Once in the sieve tubes, CMV particles interact with phloem protein 1 (PP1), and translocate jointly in the tubes. Pallas et al. [103] suggest that additional phloem proteins function in the sieve tubes that likely bind to viral particles and facilitate their transport. Any mutations within the N- or C-terminal sequences of the AMV CP blocked the systemic transport [107]. Pallas et al. [7], Tenllado and Bol [108], and Bol [107] indicate that the tissue associated with the transport of these *Bromoviridae* is most likely the phloem. Until now, there was no reports about the type of vascular tissue responsible for PDV transport. However, Kozieł et al. [32] showed that PDV CP epitope localized in companion cells, sieve tubes, but also in xylem tracheary elements, suggesting both phloem and xylem are responsible for its systemic transport.

## 7. Plant Response to Infections with PDV and Other *Bromoviridae*

Response reactions to PDV infection are mainly undescribed. One example of available information is related to various symptoms induced by different strains in plant hosts [17]. Among PDV strains/isolates, some differences were reported [22]. Nemeth [17] showed that certain PDV isolates could cause different plant diseases (Table 2), as reflected by specific viral names (Table 2). The symptoms can range from chlorotic ringspots, and necrotic changes to even gum leak in apricots.

Regardless of type of disease, PDV infection manifests as a significant decrease in fruit yield. Nemeth [17] noted that crop reduction in PDV-infected cherry cv. *Schattenmorelle* fruits reached 94–96%. Reduction of the number of cherry fruits was accompanied by 9–15% height reduction in infected trees in comparison to healthy plants. In the case of sweet cherry, the range of fruit reduction was 30–90%. Brunt et al. [2] and Kajati [14] observed, respectively, the reduction in diameter (40%) and length (35%) of PDV-infected peach shoots. Moreover, on peach trees, these authors observed 18% less leaves with 73% reduction in leaf surface. Other alternations with PDV infection include flowering disorder, increased low temperature sensitivity of flower buds, flower deformation (formed flowers had no stamens), and premature leaf fall. One of the most important reactions is associated with significant reduction of fruit yield from orchard trees. Nemeth [16] showed that budding effectiveness of different natural PDV hosts ranged from 5% to 99%.

Until now, the mechanisms of plant response to *Bromoviridae* have been only described for CMV. The effects strongly depend upon CMV gene expression at the beginning of infection. *Arabidopsis thaliana* plants (ecotype C24) were resistant to CMV strain Y (*yellow*) [103,109]. The resistance correlated with the expression of *RCY1* 104 kDa protein that belongs to the family of nucleotide-binding leucine-rich repeat proteins (CC-NBS-LRR). These proteins are responsible for signal transduction related to ethylene and salicylic acid pathways, inducing necrotic changes in the infected tissue. Necrosis occurred at the inoculation sites, localizing the virus and limiting its spread to other plant organs [109]. Inaba et al. [110] demonstrated that formation of necrosis in *Arabidopsis thaliana* was caused by the interaction of a CMV suppressor (coded by ORF2b) with a plant catalase. Alike for numerous plant viruses, CMV infection induces the RNA interference (RNAi)-based response, which is suppressed by CMV protein 2b. The interaction of RNAi response machinery with sgRNA2b triggers the silencing of CHLI, the host gene that is involved in chlorophyll biosynthesis, and thus, resulting in the yellowing of infected leaves [110].

## 8. Conclusions

Prune dwarf virus remains an enigmatic pathogen, even in the context of accumulated knowledge about other *Bromoviridae*. The available data mostly concern local transport, missing however, the information about molecular or ultrastructural effects in the infected tissue. Further studies are required for identification of cell components that contribute to PDV infection, and for characterization of pathological changes in the infected plant tissue. It is likely that the accumulating knowledge will reveal new means of resistance against PDV, one of the most dangerous plant viruses debilitating the stone fruit trees.

## Figures and Tables

**Table 1:** Taxonomy classification of *Ilarviruses* based on information from Bujarski et al. [1].

Ilarviruses	
Number	Name
Subgroup 1	
1	Parietaria mottle virus, PMoV
2	Tobacco streak virus, TSV
Subgroup 2	
3	Asparagus virus 2, AV-2
4	Citrus leaf rugose virus, CiLRV
5	Citrus variegation virus, CVV
6	Elm mottle virus, EMoV
7	Lilac ring mottle virus, LiRMoV
8	Spinach latent virus, SpLV
9	Tulare apple mosaic virus, TaMV
Subgroup 3	
10	Apple mosaic virus, ApMV
11	Blueberry shock virus, BlShV
12	Prunus necrotic ringspot virus, PNRSV
Subgroup 4	
13	Fragaria chiloensis latent virus, FCILV
14	Prune dwarf virus, PDV
No relationships to other existing groups	
15	American plum line pattern virus, APLPV
16	Humulus japonicus latent virus, HJLV

**Table 2:** Different names of diseases on *Prunus* species caused by various strains/isolates of PDV according information from Nemeth [17], modified.

Infected Species	Strain Name	Disease Name
*Prunus avium, P. cerasifera, P. cerasus, P. domestica, P. mahaleb*	Cherry chlorotic ringspot of *Prune dwarf virus*	Cherry chlorotic ringspot
*Prunus avium, P. cerasifera, P. cerasus, P. domestica, P. mahaleb*	Cherry chlorotic necrotic ringspot of *Prune dwarf virus*	Cherry chlorotic ringspot
*Prunus avium, P. cerasus*	Cherry ring mosaic of *Prune dwarf virus*	Cherry chlorotic necrotic ringspot
*Prunus avium*	Cherry ring mottleof *Prune dwarf virus*	Cherry ring mosaic
*Prunus avium*	Cherry yellow mosaic of *Prune dwarf virus*	Cherry ring mottle
*Prunus serrulata cv. Amanogawa, Prunus serrulata cv. Kwanzan, P. avium var. plena, P fontanesiana, P.incisa, P. lannesiana*	Cherry yellow mottle of *Prune dwarf virus*	Cherry yellow mosaic
*Prunus domestica*	Type strain of *Prune dwarf virus*	Cherry yellow mottle
*Prunus armeniaca, P. avium, P. cerasus*	Apricot gummosis of *Prune dwarf virus*	Chlorotic-necrotic ringspot
